# Relationship among health-related quality of life and global ancestry, clinical and socioeconomic factors in type 1 diabetes in an admixed Brazilian population

**DOI:** 10.1038/s41598-022-15138-1

**Published:** 2022-06-30

**Authors:** Rossana Sousa Azulay, Débora Lago, Glaucia Abreu Silva Santos, Maria da Glória Tavares, Vandilson Rodrigues, Marcelo Magalhaês, Roberta Ferreira Reis, Nayara Nunes, Ana Gregória Ferreira Pereira Almeida, Adriana Guimarães Sá, Gilvan Nascimento, Sabrina Damianse, Viviane Rocha, Dayse Aparecida Silva, Marília Brito Gomes, Manuel Faria

**Affiliations:** 1 Service of Endocrinology, University Hospital of the Federal University of Maranhão (HUUFMA/EBSERH), São Luís, Brazil; 2 Research Group in Clinical and Molecular Endocrinology and Metabology, Federal University of Maranhão, São Luís, Brazil; 3 DNA Diagnostic Laboratory, Rio de Janeiro State University, Rio de Janeiro, RJ Brazil; 4 Diabetes Unit, State University of Rio de Janeiro, Rio de Janeiro, Brazil

**Keywords:** Genetics, Diseases, Endocrinology

## Abstract

We aimed to evaluate the Health-related quality of life (HRQoL) of Type 1 diabetes mellitus (T1D) patients in an admixed Brazilian population. This is a cross-sectional study with 152 T1D patients. HRQoL information was obtained from two self-completed questionnaires: Short Form-6 dimensions and EuroQol-5 dimensions with visual analog scale. For inference of global ancestry, the panel of 46 autosomal informational insertion/deletion ancestry markers was used. Demographic and socioeconomic data, presence of chronic complications, glycemic control level, and type of treatment were obtained. Patients with good HRQoL were: male, under 18 years old, had health insurance, less than 5 years of diagnosis, practiced physical activity, without hypoglycemia in the last 30 days, absence of retinopathy and nephropathy, a participant in educational activities, used analogous insulin, monitoring blood glucose, observed maximum adherence to treatment and came from the secondary service. Global ancestry and self-reported color/race did not influence HRQoL indexes. Our study is the first to measure HRQoL, global ancestry and recognize the impact of T1D on the lives of patients in the State of Maranhão, Brazil. The results validate the need to provide T1D patients with continuous training on self-management and self-monitoring, aiming for better results in metabolic control and, subsequently, in the prevention of acute and chronic complications, in order to generate positive impacts on the quality of life of this population. We understand that global ancestry in a highly mixed population such as ours did not influence the HRQoL of these patients.

## Introduction

Health-related quality of life (HRQoL) is defined as the value given to life, considering the functional impairments, the social repercussions induced by the disease state, complications, and treatments. This parameter also includes the political and economic organization of the health care system and the state of health (physical, psychological, and social), damages, symptoms, or disabilities. HRQoL incorporates the aspects involved in the health-disease process and the impact of such condition on the daily life of individuals^1,2^.

Chronic diseases are the factors that most affect an individual's HRQoL, given that they permanently interfere with a person’s lifestyle and limit their productive capacity and world vision. In this context, type 1 diabetes mellitus (T1D) is considered one of the diseases that most affects an individual's HRQoL, because the related therapy requires a radical change in the person and their family’s lifestyle^3^.

T1D is a chronic disease and occurs due to autoimmune destruction of β cells, leading to insulin deficiency and a lifelong need for insulin; it may cause serious short- and long-term complications. The incidence of T1D shows accentuated geographic variation, with rates varying from 38.4 in Finland, 7.6 in Brazil, and 0.5 in Korea for every 100 thousand individuals under 15 years of age. The incidence of T1D in the city of Bauru-SP between 1986 and 2006 was 13.7/100,000^4^. This incidence increased globally at a rate of approximately 3% per year^5^.

T1D affects the psychological and emotional well-being of patients and their families^6^. The metabolic control of diabetes and the improvement of HRQoL of the patients are equally important in preventing complications^7^ From childhood onset to young adulthood, lifelong treatment, and special care are required to prevent or delay the short- and long-term complications of T1D^8^. Current guidelines and studies indicate that diabetes care should be individualized for each patient, and a healthy lifestyle can improve glycemic control and decrease the risk of complications^9^.

Race and socioeconomic differences are also implicated in T1D control. Although T1D is more frequent among White non-Hispanics when compared to Africans and Hispanics, African and Hispanic children and adolescents have worse control when compared to their White peers^10^. This study aimed to evaluate the HRQoL of T1D patients from State of Maranhão and correlated with global ancestry, clinical and socioeconomic factors.

## Methodology

### Study design

This research is a cross-sectional study conducted at the Endocrinology Service of University Hospital-Federal University of Maranhão (HU-UFMA), a reference service for the care of T1D patients in the State of Maranhão. The patients who enrolled at HU-UFMA, met the inclusion criteria, and accepted to participate in the study were admitted by reading and signing an informed consent form. The consultations were performed for 18 months starting from the date of study approval by the Research Ethics Committee of the University Hospital of the Federal University of Maranhão-CEP/HU-UFMA.

### Sample and eligibility criteria

The study population consisted of T1D patients who used the specific outpatient clinic for T1D, called tertiary care service, at the UFMA High-complexity University Hospital, other non-specific endocrinology outpatient clinics for T1D, and considered secondary care services (specialized centers). With support from a diabetes specialist assigned in São Luís, State of Maranhão, the patients were referred for interview and spontaneous data collection during their routine consultations.

Inclusion criteria were patients with T1D of both sexes, aged older than 10 years old. The diagnosis of T1D was defined according to classic clinical criteria, such as polyuria, polydipsia, polyphagia, and weight loss associated with insulin therapy since diagnosis. We excluded patients who presented in the three months prior to the history of acute infectious disease or diabetic ketoacidosis, pregnancy and lactation.

### Data collection

The T1D patients underwent a clinical demographic survey using a standardized questionnaire for data collection. The demographic data included gender, age (years), self-reported color/race, age at diagnosis (years), duration of T1D (years), level of physical activity (not including work and free-time activities), education, family income, availability of health insurance, participation in an educational group, insulin regimen used, and occurrence of hypoglycemia in the last 30 days.

The clinical variables evaluated were as follows: weight (kg), height (cm), body mass index (BMI), and blood pressure (mmHg). Glycated hemoglobin (A1c) was determined by high performance liquid chromatography (reference value: 4.0%–6.0%), and albuminuria was screened by immunoturbidimetry of random urine sample collection on three occasions. For adults, BMI was categorized as underweight (< 18.5 kg/m^2^), normal (18.5–24.9 kg/m^2^), or overweight/obesity (≥ 25 kg/m^2^) based on World Health Organization (WHO) BMI classification. For participants < 18 years old, BMI was categorized based on WHO BMI-for-age cut-off values^11^.

The HRQoL was assessed by the EuroQol questionnaire, which includes two tools: EuroQol-5 dimensions (EQ-5D) and EuroQol with visual analog scale (EQ-VAS). The first descriptively analyzes five problem dimensions (mobility, self-care, usual activities, pain and discomfort, anxiety, and depression) on a three-score scale graded 1–3 (1: “I have no problems”; 2: “I have some problems”; 3: “I have extreme problems”). The EQ-VAS (general health status) consists of an analog scale from 0 (very poor health status) to 100 (optimal health status) which was used by the patients to verify or report a value that reflects their perception of their health status^12,13^.

HRQoL was also evaluated by Short Form-6 dimensions (SF-6D), which was adapted for use in Brazil and used to describe the health status and generate utility indices derived from SF-36 items. This questionnaire comprises six domains with 4–6 levels and can generate 18,000 health states. The algorithm adapted for Brazil was used to calculate the utility scores for each health status identified after SF-6D application. The dimensions evaluated in this questionnaire were as follows: functional capacity, global limitation, social aspect, pain, mental health, and vitality. The unique SF-6D score ranges from 0 to 1 and represents the strength of an individual's preference for a particular health status, with a score of zero indicating the worst health and one implying the best^14,15^.

The fundus of the eye was examined by indirect ophthalmoscopy (EYE TECH) under the effect of topical mydriatic medication. The fundus was classified as present or absent retinopathy, using the most affected eye for diagnosis^8^.

Nephropathy diabetic (ND) was assessed by the estimated glomerular filtration rate (Chronic Kidney Disease Epidemiology Collaboration equations)^16^ and urinary albumin concentration. Microalbuminuria was defined as positive by the presence of urinary albumin in concentrations above 30 mg/L and negative at below 30 mg/L in at least two samples (normoalbuminuria reference value: < 30 mg/L; microalbuminuria: > 30 mg/L)^8,17^.

DNA extraction was performed on a peripheral blood sample using the SP QIA symphony commercial kit according to the manufacturer's guidelines (Qiagen, USA).

We analyzed global ancestry using a panel of 46 autosomal informational insertion/deletion ancestry markers (AIM-Indels), amplified in a single multiplex PCR following the protocol described by Pereira et al.^18^. The detection of polymorphisms in the generated fragments was performed by capillary electrophoresis in the ABI 3500 automatic sequencer (Life Technologies). Genotyping was achieved using GeneMapper Analysis Software v.4.1 (Life Technologies). Structure v.2.3.3 software was used to estimate ancestry.

### Statistical analysis

The data were analyzed using STATA version 16.0 (Stata Corp., College Station TX, USA) and GraphPad Prism version 8 (GraphPad Software Inc., San Diego, USA). Continuous variables were expressed as mean ± standard deviation (SD), and categorical variables were summarized as frequencies and percentages. A violin plot and a triangular plot were created displaying the distribution of the autosomal proportions of African, European and Amerindian ancestry of sample.

The Shapiro–Wilk test was used to assess the normality of continuous data. One-way analysis of variance (ANOVA) followed by Bonferroni post hoc test, Kruskal–Wallis test followed by Dunnet post hoc test and independent sample t-test were applied to analyze the data. The significance level adopted was 5%.

### Ethics approval

This study was performed in line with the principles of the Declaration of Helsinki. Approval was granted by the Research Ethics Committee of the University Hospital of the Federal University of Maranhão-CEP/HU-UFMA (protocol 23523.004536/2016-76).

### Consent to participate

Informed consent was obtained from all individual participants included in the study.

### Consent for publication

All authors have consented to submission of this article for publication.

## Results

The study sample consisted of 152 T1D patients (79 males) with a mean age of 25.1 ± 10.5 years. The most declared self-reported color/race and marital status were Brown (65.1%) and single (75.7%), respectively. The highest percentage of self-reported household income per capita income was US 192-576 (1–3 Brazilian minimum wages) (61.9%). Most of the patients reported having no health insurance (75.7%). In the total sample, 52% of the patients were managed in a secondary health care unit (Table 1).

**Table 1:** Distribution of sociodemographic data of the Type 1 diabetes patients.

Variable	n = 152	(%)
**Sex**		
Male	79	(52.0)
**Age group**		
Up to 18 years	52	(34.2)
19 years or older	100	(65.8)
**Self-reported color/race**		
White	44	(29.0)
Black	9	(5.9)
Brown	99	(65.1)
**Marital status**		
Single	115	(75.7)
Married	31	(20.4)
Divorced/ separated/ widowed	6	(3.9)
**Education level (years)**		
0–8 years	33	(21.7)
9–11 years	66	(43.4)
12 years or more	53	(34.9)
**Household income per capita (BMW)**		
Less than 1	11	(7.2)
1–3	94	(61.9)
More than 3	40	(30.9)
**Private health insurance**		
Yes	37	(24.3)
No	115	(75.7)
**Level of health care delivery**		
Secondary health care unit	79	(52.0)
Tertiary health care unit	73	(48.0)

BMW = Brazilian minimum wages per month (1 Brazilian minimum wage was R$ 954 in 2018).

Table 2 describes the clinical characteristics and information on insulin use. The most striking variables were lack of physical activity, with 54.6% of the T1D patients answered their lack of participation in physical activity of any kind, 81.6% showed HbA1c ≥ 7% (the mean was 9.05 ± 2.27), 14.3% had retinopathy, and 15.8% had albuminuria > 30 mg/L.

**Table 2:** Distribution of clinical and therapy data of the Type 1 diabetes patients.

Variable	Mean ± sd	n	(%)
**Time from diabetes diagnosis**			
< 5 years		36	(23.7)
≥ 5 years		116	(76.3)
**Age at diabetes diagnosis**			
< 5 years		18	(11.8)
5–9 years		35	(23.0)
10–18 years		63	(41.5)
≥ 19 years		36	(23.7)
**BMI category**			
Underweight		24	(15.8)
Normal		96	(63.2)
Overweight/ obesity		32	(21.0)
**Blood pressure (mmHg)**			
Diastolic	115.9 ± 18.7		
Systolic	71.1 ± 10.1		
**Physical activity (days/week)**			
0 day/week		83	(54.6)
1–2 days/week		23	(15.1)
3–7 days/week		46	(30.3)
**Hypoglycemia in the last month**			
Yes		95	(62.5)
No		57	(37.5)
**Glycated hemoglobin A1c category (HbA1c)**			
< 7%		28	(18.4)
≥ 7%		124	(81.6)
Diabetic retinopathy*			
Absent		60	(85.7)
Present		10	(14.3)
**Kidney damage**			
Absent		128	(84.2)
Present (Albuminuria > 30 mg/L)		24	(15.8)
**Diabetes support group**			
Yes		28	(18.4)
No		124	(81.6)
**Intermediate/long‐acting insulin**			
NPH		92	(60.5)
Glargine/Detemir		60	(39.5)
**Fast-acting insulin**			
Regular		70	(46.0)
Lispro/Aspart/Glulisine		63	(41.5)
No use		19	(12.5)
**Self-monitoring of blood glucose**			
Yes		124	(81.6)
No		28	(18.4)
**Adherence of insulin self-administration**			
High adherence		14	(9.2)
Medium adherence		85	(55.9)
Low adherence		53	(34.9)

± sd = standard deviation. BMI = Body mass index. *Patients with no data were excluded.

**Figure 1:**
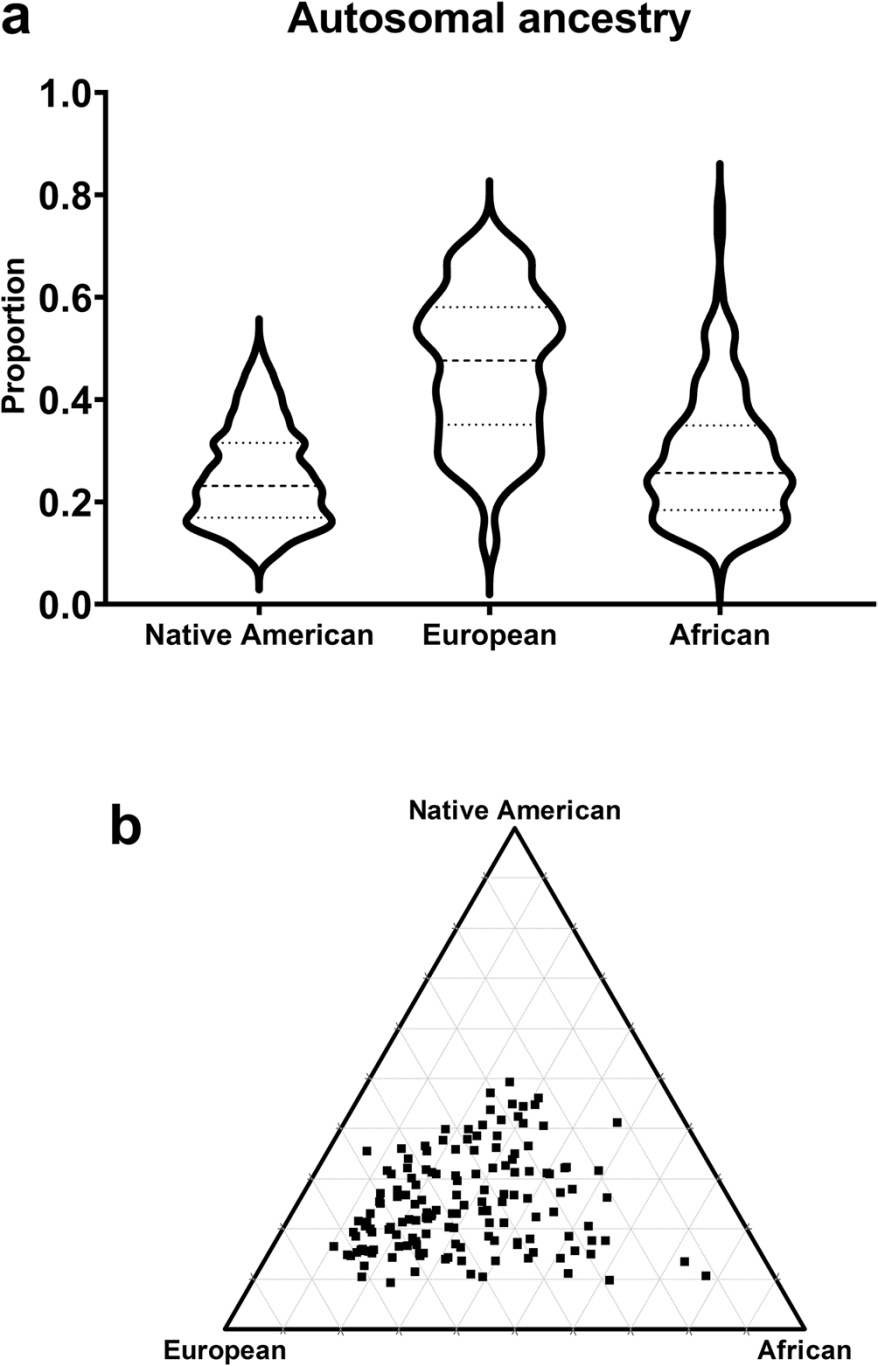
Violin plot (**a**) and triangular plot (**b**) of global ancestry in type 1 diabetes patients.

The autosomal ancestry markers showed that European ancestry was higher than African and Native American ancestries in T1D patients from Maranhão State (Fig. 1a). The ancestral proportions were 46.5 ± 14.4 European, 28.5 ± 12.7 African, and 24.9 ± 9.4 Native American. The triangular plot has shown that the T1D sample presented an admixed ancestry closer to European (Fig. 1b).

The mean scores attributed to HRQoL in the T1D patients from Maranhão State were 0.76 ± 0.18 (SF-6D), 0.90 ± 0.10 (EQ-5D), and 75.6 ± 21.2 (EQ-VAS). The association between sociodemographic variables and health-related quality of life is shown in Table 3. The SF-6D index value was statistically higher in males, up to 18 years old, private health insurance users, and secondary health care unit users. EQ-5D index value was statistically higher in males, and people up to 18 years. EQ-VAS score was higher in people up to 18 years old, and single people.

**Table 3:** Association between sociodemographic variables and health-related quality of life in type 1 diabetes patients.

Variable	SF–6D	*P* value	EQ–5D	*P* value	EQ–VAS	*P* value
	Mean ± sd		Mean ± sd		Mean ± sd	
**Sex**		**0.005**		**0.009**		0.215
Male	0.80 ± 0.17		0.92 ± 0.10		77.7 ± 20.8	
Female	0.71 ± 0.19		0.88 ± 0.11		73.4 ± 21.5	
**Age group**		**0.001**		**0.006**		**0.001**
Up to 18 years	0.83 ± 0.16		0.93 ± 0.08		83.0 ± 18.6	
19 years or older	0.72 ± 0.19		0.88 ± 0.11		71.8 ± 21.5	
**Self-reported color/race**		0.658		0.089		0.229
White	0.76 ± 0.16		0.92 ± 0.09		73.8 ± 20.3	
Black	0.70 ± 0.21		0.83 ± 0.08		65.6 ± 18.6	
Brown	0.76 ± 0.19		0.90 ± 0.11		77.3 ± 21.7	
**Marital status**		**0.001**		0.243		**0.005**
Single	0.78 ± 0.18ª		0.91 ± 0.09		78.6 ± 19.3ª	
Married	0.71 ± 0.16^ab^		0.86 ± 0.14		68.0 ± 22.5^b^	
Divorced/separated/widowed	0.53 ± 0.23^b^		0.87 ± 0.13		58.3 ± 33.1^b^	
**Education level (years)**		0.355		0.864		0.312
0–8 years	0.78 ± 0.22		0.91 ± 0.10		83.0 ± 21.7	
9–11 years	0.74 ± 0.19		0.90 ± 0.10		74.2 ± 22.5	
12 years or more	0.77 ± 0.15		0.90 ± 0.10		72.8 ± 18.4	
**Head of the family**		0.945		0.921		0.884
Yes	0.76 ± 0.18		0.90 ± 0.12		76.1 ± 19.5	
No	0.76 ± 0.19		0.90 ± 0.10		75.5 ± 21.8	
**Household income per capita (BMW)**		0.605		0.716		0.338
Less than 1	0.70 ± 0.19		0.89 ± 0.11		80.0 ± 15.4	
1 to 3	0.76 ± 0.20		0.91 ± 0.11		76.9 ± 22.0	
More than 3	0.76 ± 0.16		0.89 ± 0.09		72.0 ± 20.6	
**Private health insurance**		**0.025**		0.208		**0.028**
Yes	0.81 ± 0.19		0.92 ± 0.09		82.7 ± 14.9	
No	0.74 ± 0.18		0.89 ± 0.11		73.4 ± 22.4	
**Level of health care delivery**		**0.032**		0.990		**0.048**
Secondary health care unit	0.79 ± 0.18		0.90 ± 0.11		78.9 ± 21.2	
Tertiary health care unit	0.72 ± 0.18		0.90 ± 0.09		72.1 ± 20.8	

SF–6D = Short-Form–6 dimensions. EQ–5D = European quality of life–5 dimensions. EQ–VAS = European quality of life–visual analog scale. ± sd = standard deviation. BMW = Brazilian minimum wages per month (1 Brazilian minimum wage was R$ 954 in 2018). Bold indicates *P* values < 0.05 (one-way ANOVA, Kruskal–Wallis, independent sample t-test or Mann–Whitney test). Different letters indicate significant differences between categories using Bonferroni's or Dunnett's multiple comparison test (*P* values < 0.05).

Table 4 shows the association between clinical variables and health-related quality of life. The SF-6D index was statistically higher in patients with from diabetes diagnosis ≥ 5 years. Physical activity 3 or more days a week and no hypoglycemic episodes in the past month were associated with higher SF-6D and EQ-5D scores. Patients who present retinopathy had lower SF-6D and EQ-VAS indexes. The SF-6D index was lower in patients who had albuminuria. Patients who participated in the diabetes education group and using Glargine/Detemir use and Lispro/Aspart/Glulisine presented higher HRQoL indexes. T1D patients who performed self-monitoring of blood glucose had higher EQ-5D and EQ-VAS indexes.

**Table 4:** Association between clinical variables and health-related quality of life in type 1 diabetes patients.

Variable	SF–6D	*P* value	EQ–5D	*P* value	EQ–VAS	*P* value
	Mean ± sd		Mean ± sd		Mean ± sd	
**Time from diabetes diagnosis**		**0.048**		0.588		0.315
≥ 5 years	0.81 ± 0.17		0.92 ± 0.08		78.7 ± 23.3	
< 5 years	0.74 ± 0.19		0.90 ± 0.11		74.6 ± 20.5	
**Age at diabetes diagnosis**		0.975		0.233		0.236
< 5 years	0.75 ± 0.16		0.89 ± 0.10		78.3 ± 18.3	
5–9 years	0.77 ± 0.19		0.93 ± 0.09		81.4 ± 18.6	
10–18 years	0.76 ± 0.20		0.88 ± 0.12		73.4 ± 22.4	
≥ 19 years	0.76 ± 0.17		0.91 ± 0.07		72.6 ± 22.2	
**BMI category**		0.736		0.177		0.088
Underweight	0.78 ± 0.21		0.93 ± 0.09		82.9 ± 19.2	
Normal	0.76 ± 0.18		0.90 ± 0.11		75.6 ± 20.6	
Overweight/obesity	0.74 ± 0.18		0.89 ± 0.07		70.3 ± 23.2	
**Physical activity (days/week)**		**0.023**		**0.013**		0.216
0 day/week	0.72 ± 0.19ª		0.88 ± 0.12ª		73.3 ± 22.1	
1–2 days/week	0.81 ± 0.18^ab^		0.94 ± 0.09^ab^		81.9 ± 17.1	
3–7 days/week	0.80 ± 0.16^b^		0.92 ± 0.07^b^		76.6 ± 21.1	
**Hypoglycemia in the last month**		**0.002**		**0.024**		0.464
Yes	0.72 ± 0.19		0.89 ± 0.10		74.6 ± 20.8	
No	0.82 ± 0.15		0.92 ± 0.11		77.2 ± 21.9	
**Glycated hemoglobin A1c category**		0.514		0.266		0.684
< 7%	0.78 ± 0.19		0.92 ± 0.08		77.1 ± 24.4	
≥ 7%	0.75 ± 0.18		0.90 ± 0.11		75.3 ± 20.5	
**Diabetic retinopathy**		**0.024**		0.127		**0.018**
Absent	0.73 ± 0.18		0.90 ± 0.11		78.3 ± 17.7	
Present	0.61 ± 0.14		0.83 ± 0.17		61.5 ± 32.4	
**Kidney damage**		**0.018**		0.144		0.105
Absent	0.78 ± 0.18		0.91 ± 0.10		77.3 ± 19.8	
Present (Albuminuria > 30 mg/L)	0.70 ± 0.20		0.88 ± 0.12		71.0 ± 24.5	
**Diabetes support group**		**0.021**		**0.027**		**0.001**
Yes	0.83 ± 0.17		0.94 ± 0.07		86.9 ± 13.9	
No	0.74 ± 0.18		0.89 ± 0.11		73.1 ± 21.8	
**Intermediate/long‐acting insulin**		**0.002**		**0.045**		**0.001**
NPH	0.72 ± 0.19		0.89 ± 0.11		71.0 ± 22.9	
Glargine/Detemir	0.82 ± 0.17		0.92 ± 0.08		82.6 ± 16.0	
**Fast-acting insulin**		** < 0.001**		**0.009**		**0.028**
Regular	0.69 ± 0.18ª		0.88 ± 0.11ª		71.2 ± 23.2ª	
Lispro/Aspart/Glulisine	0.82 ± 0.15^b^		0.93 ± 0.07^b^		80.5 ± 16.7^b^	
No use	0.82 ± 0.20^b^		0.90 ± 0.14^ab^		74.4 ± 23.8^ab^	
**Self-monitoring of blood glucose**		0.091		**0.003**		** < 0.001**
Yes	0.77 ± 0.18		0.92 ± 0.08		79.2 ± 17.5	
No	0.70 ± 0.20		0.84 ± 0.15		59.6 ± 28.2	
**Adherence of insulin self-administration**		0.462		0.688		0.060
High adherence	0.77 ± 0.23		0.92 ± 0.11		77.8 ± 30.1	
Medium adherence	0.77 ± 0.18		0.90 ± 0.10		78.0 ± 19.5	
Low adherence	0.73 ± 0.18		0.89 ± 0.11		71.3 ± 20.8	

SF–6D = Short-Form–6 dimensions. EQ–5D = European quality of life–5 dimensions. EQ–VAS = European quality of life–visual analog scale. ± sd = standard deviation. BMI = Body mass index. Bold indicates *P* values < 0.05 (one-way ANOVA, Kruskal–Wallis, independent sample t-test or Mann–Whitney test). Different letters indicate significant differences between categories using Bonferroni's or Dunnett's multiple comparison test (*P* values < 0.05).

The relationship between global ancestry and HRQoL was analyzed (Table 5). The proportion of Native America, European and African ancestries were categorized as terciles. The global ancestry terciles showed no significant differences in HRQoL indexes.

**Table 5:** Association between global ancestry and health-related quality of life in type 1 diabetes patients.

Global ancestry	SF–6D	*P* value	EQ–5D	*P* value	EQ–VAS	*P* value
	mean ± sd		mean ± sd		mean ± sd	
**Native American**		0.256		0.554		0.305
1st tertile	0.79 ± 0.20		0.91 ± 0.09		79.4 ± 20.9	
2nd tertile	0.73 ± 0.21		0.89 ± 0.11		73.4 ± 19.1	
3rd tertile	0.76 ± 0.14		0.90 ± 0.10		74.2 ± 23.1	
**European**		0.919		0.524		0.294
1st tertile	0.75 ± 0.17		0.89 ± 0.10		71.7 ± 23.9	
2nd tertile	0.76 ± 0.19		0.90 ± 0.11		77.6 ± 19.8	
3rd tertile	0.76 ± 0.19		0.91 ± 0.09		77.4 ± 19.7	
**African**		0.642		0.510		0.805
1st tertile	0.77 ± 0.18		0.91 ± 0.09		77.1 ± 19.2	
2nd tertile	0.76 ± 0.17		0.90 ± 0.10		74.3 ± 23.4	
3rd tertile	0.74 ± 0.20		0.89 ± 0.11		75.5 ± 21.0	

SF–6D = Short-Form–6 dimensions. EQ–5D = European quality of life–5 dimensions. EQ–VAS = Europen quality of life–visual analog scale. ± sd = standard deviation. *P* values calculated by one-way ANOVA or Kruskal–Wallis test.

In addition, significant differences were found in proportion of European and African ancestries according to self-reported color/race (*P* < 0.001). Self-reported Whites had higher European ancestry, and self-reported Blacks had higher African ancestry. A statistically higher European ancestry was observed in patients with glycated hemoglobin A1c ≥ 7% (*P* = 0.035). Diabetic retinopathy and kidney damage had no association with proportion of global ancestry in the evaluated sample (Table 6).

**Table 6:** Proportions of African, European and Native American ancestry according to self-reported race/color and clinical outcomes.

Global ancestry	Native American	*P* value	European	*P* value	African	*P* value
	mean ± sd		mean ± sd		mean ± sd	
**Self-reported color/race**		0.303		** < 0.001**		** < 0.001**
White	0.24 ± 0.09		0.53 ± 0.13ª		0.22 ± 0.10^a^	
Black	0.20 ± 0.11		0.29 ± 0.11^b^		0.50 ± 0.14^b^	
Brown	0.25 ± 0.09		0.45 ± 0.13^c^		0.29 ± 0.11^c^	
**Glycated hemoglobin A1c**		0.095		**0.035**		0.210
< 7%	0.27 ± 0.10		0.41 ± 0.14		0.31 ± 0.15	
≥ 7%	0.24 ± 0.09		0.47 ± 0.14		0.27 ± 0.12	
**Diabetic Retinopathy**		0.075		0.262		0.937
Absent	0.23 ± 0.08		0.48 ± 0.15		0.28 ± 0.14	
Present	0.28 ± 0.10		0.42 ± 0.15		0.28 ± 0.10	
**Kidney damage**		0.659		0.310		0.410
Absent	0.24 ± 0.09		0.47 ± 0.14		0.28 ± 0.13	
Present	0.25 ± 0.08		0.44 ± 0.15		0.30 ± 0.11	

± sd = standard deviation. *P* values calculated by one-way ANOVA. Different letters indicate significant differences between categories using Bonferroni’s multiple comparison test (*P* values < 0.05).

## Discussion

This study is the first to assess the quality of life of patients with T1D in the State of Maranhão, an admixed population of the Northeast region of Brazil. In our evaluation, the variables that positively influenced the HRQoL of these patients included the following: being male, age under 18 years old, single status, elementary school education, having health insurance, having less than five years of diagnosis, and practicing certain types of physical activity. In addition, non-occurrence of hypoglycemia in the last 30 days, lack of chronic complications (retinopathy and nephropathy), participation in any group educational activity, using analogous insulin, monitoring blood glucose, maximum adherence to treatment, and coming from secondary service showed statistical significance. Global ancestry and self-reported color/race did not show influence on HRQoL indexes.

In a multicenter national study conducted in 2015, the average score attributed to general health (EQ-VAS) was lower than that obtained in the present study (72.5 ± 22 vs. 76.24 ± 21.30). The differences found in the literature in relation to the quality of life are related to urbanization, poverty, and organization of health services. However, despite this structure, the study mentioned above showed better quality of life reported in the EQ-VAS (74.6 ± 30), lower depression rate, and lower anxiety frequency in the northeast region, which characteristically features a lower urbanization rate, wealth, and structuring in health services. These findings were like those observed in our study, suggesting additional factors, such as lifestyle, in this assessment^19^.

When we associated the socio-demographic criteria with the quality of life through HRQoL, the male patients showed better HRQoL. Other studies have already observed the lower quality of life among female patients^3,20,21^. Women generally present higher disease-related concerns, lower level of satisfaction, and worse perception of their health compared with men^21^.

We observed significant improvement in the HRQoL in the group with supplementary health insurance. The American Diabetes Association observed that patients with public and private health insurance have more access to health care and thus achieve better glycemic indexes and quality of life^9^. A recent study evaluating a significant sample of the Brazilian population corroborates our findings of fewer diabetes-related chronic complications, especially in retinopathy, when patients have access to public and private health services^22^.

We also observed better HRQoL in the patients who participated in group education in secondary care. A study in the United Kingdom evaluated the HRQoL before and after a three-day educational course offered to adolescents with T1D to help manage diabetes. The results showed that the group's pre-course yearnings were met, and the educational assessment was solid. The A1c indices and BMI were unchanged, and no episodes of hypoglycemia were observed; both parents and patients reported an improved HRQoL after training^23^. In a Greek study, patients with T1D participated for 1 year in groups, in which knowledge about the disease was transmitted in a simple and understandable manner. At the end of this program, reduced A1c levels, fewer blood glucose fluctuations, and lower incidence of hypoglycemia were observed, improving the HRQoL of these patients^24^.

The use of analog insulin also demonstrated a positive impact on the HRQoL of our population. In the literature, this finding is controversial. A 2018 meta-analysis showed no overall difference in HRQoL compared with the use of the recombinant human insulin (NPH)^25^. A Brazilian meta-analysis in 2019 failed to reach a consensus on the superiority of using fast acting analogs over regular insulin due to the scarcity of well-designed studies in the literature^26^. However, reductions in severe hypoglycemia, postprandial blood glucose, and HBA1c, factors that impact the quality of life, were observed.

HBA1c is an indirect measure of mean blood glucose levels, reflecting blood glucose levels over the past three months; despite its known limitations, it remains the primary tool for ensuring glycemic control and predicting the risk of complications^27^. The mean HBA1c in this study was slightly lower than that observed in a multicenter Brazilian study in 2015, specifically in terms of the overall average of Brazil (9.05 ± 2.27 vs. 9.4 ± 2.4) and in the evaluation of the northeast region of Brazil (9.05 ± 2.27 vs. 9.6 ± 2.6). No significant difference in HRQoL was observed between the patients with or without good metabolic control as evaluated by this tool. We attribute this finding to the large number of patients without good metabolic control, that is, 82.39% of our population. In a cohort from the diabetes outpatient clinic of the Hospital das Clinicas of the Federal University of Paraná with T1D adolescents, the patients with the best HRQoL included those with lower HBA1c levels; the higher the HBA1c, the greater the likelihood of lower levels of satisfaction^28^. A previous study assessing adolescents with T1D observed that those with A1c in their goal may realize that diabetes results in an unfavorable effect on their lives, resulting in depression and difficulty in coping with the disease^29^.

Physical activity is related to patients' lifestyle and is an impact variable in diabetes care. Every patient should be encouraged to allot leisure periods of physical activity and balanced exercise^9^. In this study, sedentary lifestyle exhibited a significant negative impact on the HRQoL, in line with other studies conducted on patients with T1D and HRQoL^20,28^. Physical activity is strongly associated with psychological well-being and therefore, should be encouraged in this population^30^.

Hypoglycemia, another important factor in the evaluation of this group, is described as the main limiting factor in the management of T1D^9^. The data obtained in our research regarding the frequency hypoglycemia episodes and the fear of these episodes are in line with other studies demonstrating impairment in HRQoL^31^. Hypoglycemia has been associated with cognitive dysfunction in children with T1D, and the fear of hypoglycemia may add to the difficulty faced by patients in adhering to the proposed treatment and therefore glycemic control^32,33^.

According to the literature, insufficient diabetes control and increased BMI negatively influence HRQoL, because they generate emotional disorders, such as anxiety, anguish, depression, low self-esteem, anorexia, or bulimia, whereas adequate capillary blood glucose monitoring and dietary flexibility are related to higher levels of HRQoL^21^. We observed no difference in the HRQoL between the patients with different BMI levels. However, capillary blood glucose monitoring showed improvement in the HRQoL of patients.

A survey of young Germans comparing the HRQoL of T1D adolescents aged 11 to 17 years old and their healthy peers showed that the diagnosis of the disease in early childhood caused no impairment in the HRQoL compared with that of the peers without diabetes. This finding is probably due to the process of adaptation common in individuals with chronic diseases, with those who experienced such process from an early age becoming accustomed to their condition, considering their disease as normal and as a part of their daily life; as a result, and individuals with chronic diseases feel no different from their healthy peers^34^. A Brazilian study showed by linear regression that complications and time of diabetes had low impact on EQ-VAS and failed to clarify the causes^18^. Our study failed to show a better HRQoL of patients when considering the same variables. With earlier diagnosis of T1D, a better EQ-VAS was observed in patients with less disease time, and this finding is related to the absence of complications.

The presence of microvascular complications (retinopathy and nephropathy) was associated with a lower HRQoL in our study. The 23-year study of DCCT/DTIS also showed that the presence of microvascular complications significantly decreased the HRQoL in patients with T1D^35^. Retinopathy and nephropathy impair the autonomy, self-care, and HRQoL of patients with T1D^36^.

Our study has shown a better HRQoL in patients coming from the secondary public service, which can be attributed to the higher number of patients using insulin analogs and presenting less hypoglycemia, which are important factors affecting the HRQoL of our patients. In addition, these patients had health insurance and received various services in private services, which may also influence their perception of HRQoL. These patients also had more access to educational programs, which have been shown by several studies to positively impact HRQoL^23,24^.

We found a negative correlation between European ancestry and glycemic control, the higher the degree of European ancestry the worse HBA1c. Evidence suggests that minority populations tend to have poorer self- management and diabetes outcomes, for example African and Hispanic children and adolescents have worse control when compared to their White peers^10,37^. We hypothesize that the difference in our study is due to the large percentage of European ancestry found in our highly mixed population.

Our population is composed by the miscegenation between European, African, and Native American populations^38^. This fact was noted in our study through the analysis of genomic ancestry. Through our analyses we found that, as in all Brazilian regions, European ancestry was the largest contributor, but in our population, it approached 50% differing from the weighted average of 68.1% found in the Brazilian population in a systematic review study conducted in 2019. We also obtained a similar percentage between African and Native American ancestry (around 25% each), which again differs from the Brazilian average of 19.6% African and 11.6% Native American. The distribution of ancestral groups did not occur homogeneously in the Brazilian territory, differing depending on the geographic region, and reflecting the history of colonization with different levels of miscegenation^39^. In general, this occurred with an asymmetrical mating pattern, preferably between European men and Native American or African women. In Afro-descendant communities such as the Amazon and Maranhão, another pattern between African men and Native American women was also observed^38^. Also, in Maranhão, Native Americans maintained contact with the Brazilian population of mixed race and with African slaves, in the pattern of the mating of Native American men with African or mixed women^40^. These patterns may justify our observed ancestry panel.

Surveys have shown a low correlation between color report and ancestry, however, in our analysis we found that in self-reported Whites had higher European ancestry, and self-reported Blacks had higher African ancestry, suggesting adequate perception by our patients^41^.

The present study has some limitations. For the diagnosis of T1D, we did not use the dosage of autoantibodies and C-peptide levels, but as aforementioned, we used the classic clinical diagnostic criteria of the disease. We also consider the reduced size of our sample to be a limitation, which may impact the lack of association between HRQoL indices and some factors such as global ancestry proportion and BMI. In addition, the current cross-sectional design was unable to determine the direction of causality. Thus, longitudinal studies with a larger sample and other biomarkers may further clarify these relationships in T1D population.

## Conclusion

Quality of life questionnaires are barely explored in Brazil, particularly in the State of Maranhão, the northeast region of the country. Our study is the first to measure HRQoL and recognize the impact of T1D through the analysis of multiple factors related to the quality of life and global ancestry in patients with T1D from Maranhão, a state formed by a population highly admixed. We understand that ancestry in a highly mixed population such as ours did not influence the quality of life of these patients, however research with larger samples is necessary to clarify this impression. The results validate the need to provide T1D patients with continuous training on self-management and self-monitoring, seeking better results in metabolic control and consequently, in the prevention of acute and chronic complications to generate positive impacts on the quality of life of this population. In addition, reinforcing physical activity at each appointment should be part of the health team’s routine.

## Data Availability

All data generated or analyzed during this study are included in this published article.
